# Chemical Profile of the Organic Residue from Ancient Amphora Found in the Adriatic Sea Determined by Direct GC and GC-MS Analysis

**DOI:** 10.3390/molecules16097936

**Published:** 2011-09-14

**Authors:** Igor Jerković, Zvonimir Marijanović, Mirko Gugić, Marin Roje

**Affiliations:** 1 Faculty of Chemistry and Technology, University of Split, N. Tesle 10/V, 21000 Split, Croatia; 2 University of Applied Sciences Marko Marulić in Knin, P. Krešimira IV 30, 22300 Knin, Croatia; 3 Department of Organic Chemistry, Laboratory for Stereoselective Catalysis and Biocatalysis, Ruđer Bošković Institute, Bijenička cesta 54, 10000 Zagreb, Croatia

**Keywords:** amphora organic residue, HS-SPME, GC-MS, methyl dehydroabietate, retene

## Abstract

An ancient organic residue was collected from the bottom of a Greco-Italian amphora found in the Adriatic Sea and investigated by direct GC and GC-MS analysis. The headspace composition was determined by HS-SPME using: (1) DVB/CAR/PDMS and (2) PDMS/DVB fibres. Higher percentages of benzene derivatives, monoterpenes and other low-molecular aliphatic compounds were obtained by method (1) in contrast to higher percentage of naphthalene and phenanthrene derivatives found by method (2). In comparison with the composition of pine resin, it is more likely that the found low-molecular aliphatic alcohols, acids, esters and carbonyls with 2-phenylethanol were trapped and preserved within the organic residue from stored wine – the amphora’s originally content. Semi-volatile diterpenes methyl dehydroabietate (33.6%) and retene (24.1%) were dominant in the residue CH_2_Cl_2_ solution. Other abundant compounds were 1,4-dimethoxyphenanthrene (6.8%) as well as other naphthalene and/or phenanthrene derivatives [7-(1-methylethyl)-1,4a-dimethyl-1,2,3,4,4a,9,10,10a-octahydronaphthalene, 7-(1-methylethyl)-1,4a-dimethyl-2,3,4,4a,9,10-hexahydrophenanthrene, 7-(1-methylethyl)-1,4a-dimethyl-1,2,3,4,4a,9,10,10a-octahydro-phenanthrene, 3,6-dimethylphenanthrene and 2,3,5-trimethylphenanthrene]. Possible sources and formation pathways of the major compounds in the residue were discussed.

## 1. Introduction

The amphora is one of the most characteristic products of the ancient Mediterranean area. Globular forms of amphorae usually carried oil (*Amphorae olearie*). Wine was carried in amphorae of distinctive long shape (*Amphorae vinariae*) that were often coated with a thin layer derived usually from pine resin or resinous wood [1]. Due to its hydrophobic properties this resinous coat acted as a water-proofing agent sealing the inside of the amphorae and gave the wine a special flavor.

Two main types of sealing material were used for archaeological amphorae [2]: wood tar and pitch. Wood tar was produced from the dry distillation of resinous wood in stone ovens [3]. Pitch was obtained by heating the resin to separate the most volatile terpene compounds (turpentine) [1]. Wood tar and pitch have a very similar chemical composition, basically diterpenes, depending on the starting materials, the temperature to which they were heated and the conditions of preservation [4]. The presence or absence of methyl esters has been considered as an indicator to distinguish the material produced from wood [2,5].

Concerning their nature and composition, the chemistry of natural resins exuded by trees is diverse, but most are composed of terpenes made up of isoprene units [6,7]. In general, the organic residue analysis involves extraction of the compounds that are either absorbed within the ceramic matrix or preserved within visible encrustations on the surface. Different techniques have been applied for the identification of diterpenoid compounds and food traces in the organic residues such as TLC, HPLC, IR and GC [8,9,10,11]. GC analysis of the residue polar high-molecular compounds is often performed after saponification, extraction and derivatization by silylation, methylation or alkyl chloroformate reactions [12]. Each of these techniques has some drawbacks: silylation must be performed under anhydrous conditions and requires heating of the sample and injection of reactive mixtures onto the GC column. Methylation of acids with diazometane is easy to perform, but side-products are obtained. The ratios among solvent components have to be optimized for alkyl chloroformates in order to reduce side-product formation.

The aim of this work is to determine the chemical profile of the headspace and volatile organic compounds (VOCs) of the rare resinous organic deposit obtained from an ancient Roman amphora found in the Adriatic Sea. A review of the relevant literature revealed that previous research on other aged organic residues were mainly focused on the determination of polar high-molecular weight compounds, neglecting the possible presence of low-molecular weight volatiles. Therefore direct analyses of the residue dissolved in CH_2_Cl_2_ (without derivatization) and exposed to headspace solid-phase microextraction (HS-SPME) were applied in this research, followed by GC and GC-MS analyses. Once the VOCs have been identified, their probable source or sources will be discussed and valuable bio-molecular information about the amphorae’s originally content could be obtained. The obtained results will be compared to the papers reporting identification of resinous deposit components of amphorae from other regions.

## 2. Results and Discussion

A total of 79 headspace and volatile compounds of the organic residue found at the bottom of an ancient Roman amphora were identified by direct GC and GC-MS analyses (without any derivatization procedure). The obtained chemical profiles of the headspace and dissolved residue are remarkably different (Table 1), but complementary for understanding the sample complexity. The headspace composition, besides valuable evidence on the monoterpene traces present, provided additionally a snapshot of trapped wine aroma traces (in comparison to fresh pine resin headspace composition). On the other hand, the chemical profile of the dissolved residue afforded essential information for understanding the ancient procedures of water-proof coating preparation. However we must keep in mind that the identified compounds probably underwent some degree of hydrolysis, oxidation, or microbial breakdown over the period of archaeological deposition. The dark brown colour of the organic residue indicates a probable and ubiquitous heating of pine resin/wood during preparation of the amphorae coating which can cause three probable effects: (1) terpene volatilization, (2) thermal dehydrogenation and (3) decarboxylation of part of the abietic acid. The residue was insoluble in water and soluble in acetone, methanol and dichloromethane. It has a resinous smell when heated and burns when in contact with a flame.

### 2.1. The Headspace Chemical Composition of the Residue (HS-SPME)

**Figure 1:**
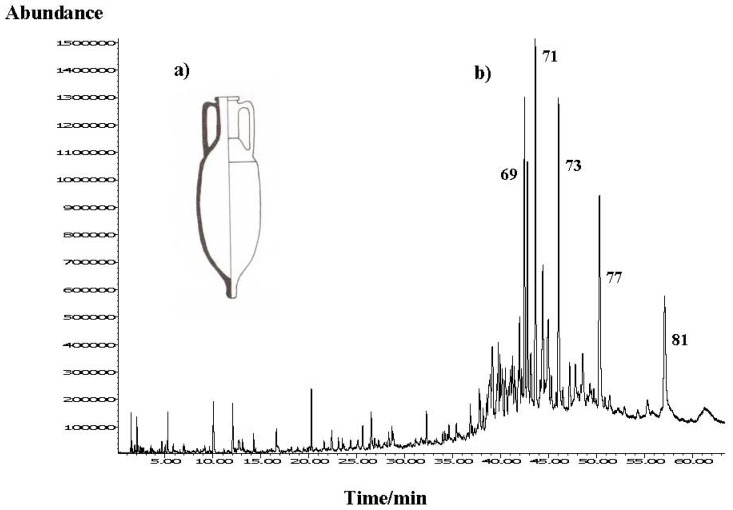
(**a**) Scheme of the Greco-Italian amphora type Benoit Republicane-II/Lamboglia with the section representing the coating. (**b**) Total ion chromatogram of the organic residue headspace composition obtained by HS-SPME (DVB/CAR/PDMS fibre) on HP-5MS column. Numbers refer to Table 1.

**Figure 2:**
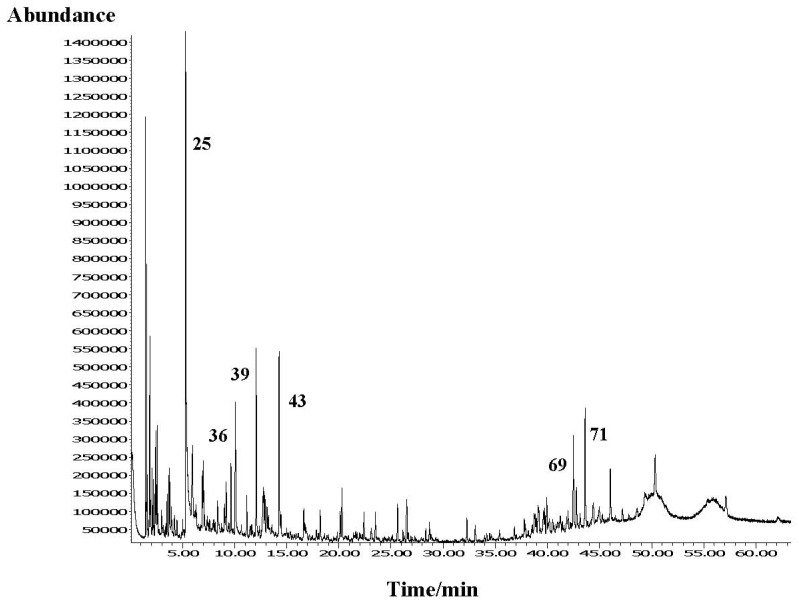
Total ion chromatogram of the organic residue headspace composition obtained by HS-SPME (PDMS/DVB fiber) on HP-5MS column. Numbers refer to Table 1.

Two fibres of different polarity were used for obtaining more detailed headspace composition of the sample. Obtained chemical profiles (Table 1) are dominated by low-molecular weight and mostly volatile compounds that were probably dissolved, trapped and consequently preserved in the internal residue. These profiles are dependent on the type of used fibre: higher percentages of benzene derivatives, monoterpenes and other low-molecular weight compounds were obtained by DVB/CAR/PDMS fibre (Figure 1) in contrast to the higher percentage of naphthalene and/or phenanthrene derivatives found by using PDMS/DVB fibre (Figure 2). Significantly lower presence of high-molecular weight organic compounds in the headspace was expected, since HS-SPME is not an adequate method for their analysis.

In general, monoterpenes appear as predominant components in the natural pine resin, especially α-pinene [5,6]. In the investigated sample of pine resin, the headspace percentage of α-pinene ranged from 66.2 to 73.4% (Table 2). However, the majority of monoterpenes were probably lost during heat treatment and preparation of the amphora coating due to relatively high volatility. Despite heat treatment and conditions of preservation of the ancient organic residue, several monoterpenes were only found in the headspace with minor percentages such as α-pinene (0.1–0.2%), camphene (0.2–0.4%), *p*-cymene (0.1–2.7%), limonene (0.0–0.5%), camphor (0.0–0.9%), borneol (1.1–4.5%) and bornyl acetate (0.0–0.6%). All these compounds are found in natural pine resin with considerably more headspace abundance (Table 2). In addition, the headspace composition contained a variety of low-molecular weight aliphatic carboxylic acids and esters [acetic acid (0.1–2.0%), ethyl acetate (0.0–0.4%), propionic acid (0.1–2.1%), ethyl propionate (0.0–0.4%), butanoic acid (0.0–1.6%), isovaleric acid (0.0–0.4%), valeric acid (0.0–1.2%), hexanoic acid (0.1–4.6%), heptanoic acid (0.0–0.8%), octanoic acid (0.5–1.4%) and nonanoic acid (0.0–0.5%)]; and aliphatic alcohols and carbonyl compounds [acetone (0.3–2.8%), butan-2-one (0.0–0.6%), isoamyl alcohol (0.3–0.9%), heptan-2-one (0.0–1.0%), heptanal (0.0–0.4%), octanal (0.0–0.7%), nonan-2-one (0.0–1.3%)]. In addition, several volatile benzene derivatives were found only in the headspace such as benzaldehyde (0.6–20.4%), 2-hydroxybenzaldehyde (0.0–0.2%), 1-phenylethanone (0.0–0.7%), 2-phenylethanol (1.2–3.3%) and others (Table 1).

The presence of a variety of highly-volatile aliphatic compounds with low abundance is an interesting observation since they are not present in the headspace of natural pine resin (Table 2) as well as in natural resins of other origins [5,6]. It is more likely that they were preserved within the organic residue structure as the consequence of the stored wine – the amphorae’s originally content. Several hundred compounds have been identified as important contributors to wine aroma [13]. These components comprise several classes of organic compounds, including esters, alcohols, organic acids, ketones, aldehydes, and monoterpene alcohols. Good results for wine esters, alcohols, and terpenes were obtained by HS-SMPME, but organic acids were not effectively analyzed [14,15]. Isobutanol, isoamyl alcohol, 2-phenylethanol and propan-1-ol are known secondary products of yeast metabolism. These compounds could be synthesized by yeast through either the anabolic pathway from glucose, or the catabolic pathway from their corresponding amino acids (valine, leucine, isoleucine and phenylalanine). Acetate esters are the result of the reaction of acetyl-CoA with alcohols formed by degradation of amino acids or carbohydrates. Any ancient ethanol would have been metabolized by microorganisms. Tartaric acid, a grape-wine marker of archaeological samples [16,17], was not found, nor in the CH_2_Cl_2_ solution of the sample. As an extremely water soluble compound it is thus prone to leaching and the precise method for its GC determination includes a silylation procedure [16]. Syringic acid, which is a slightly less specific biomarker for wine [18], was not detected either, but syringaldehyde was found (0.2–0.4%). Constituents of grape wine were found in other ancient samples [19] including alcohols, acids, esters, aldehydes, fatty acid derivatives, and terpenes.

Derivatives of phenanthrene were also identified, but their abundance in the sample is not reliably represented in the headspace due to their low volatility. The most abundant were: 7-(1-methylethyl)-1,4a-dimethyl-1,2,3,4,4a,9,10,10a-octahydronaphthalene (2.7–9.3%), 7-(1-methylethyl)-1,4a-dimethyl-2,3,4,4a,9,10-hexahydro-phenanthrene (1.0–7.7%), 7-(1-methylethyl)-1,4a-dimethyl-1,2,3,4,4a,9,10,10a-octahydrophenanthrene (3.4–12.2%), 3,6-dimethylphenanthrene (1.0–6.6%), 1,4-dimethoxyphenanthrene (1.7–10.0%), 1-methyl-7-(1-methylethyl)-phenanthrene (1.6–9.9%) and methyl dehydroabietate (1.0–6.6%).

### 2.2. The Residue CH_2_Cl_2_ Solution Chemical Composition 

**Figure 3:**
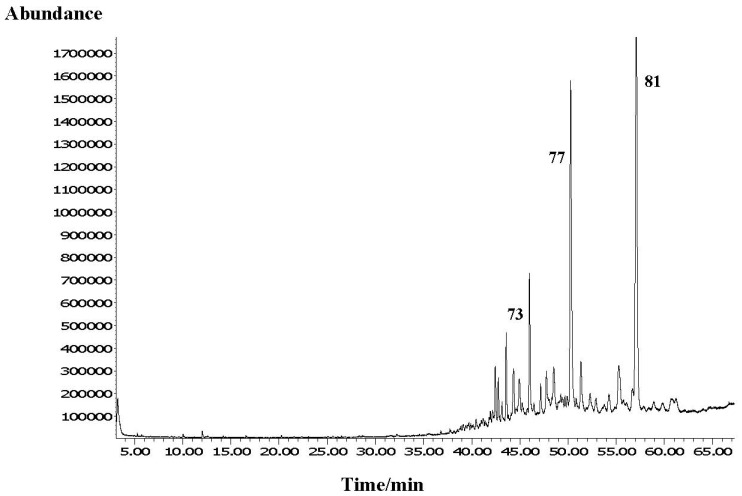
Total ion chromatogram of the organic residue solution in CH_2_Cl_2_ on HP-5MS column. Numbers refer to Table 1.

Results of GC and GC-MS analyses of the residue CH_2_Cl_2_ solution reveals the high-molecular weight diterpenes methyl dehydroabietate (33.6%) and retene (24.1%) as the major constituents, as seen in other ancient organic residues [2,3]. A representative chromatogram is presented in Figure 3.

Other abundant compounds were 1,4-dimethoxyphenanthrene (6.8%) as well as naphthalene and phenanthrene derivatives [7-(1-methylethyl)-1,4a-dimethyl-1,2,3,4,4a,9,10,10a-octa-hydronaphthalene (2.8%), 7-(1-methylethyl)-1,4a-dimethyl-2,3,4,4a,9,10-hexahydrophenanthrene (1.9%), 7-(1-methylethyl)-1,4a-dimethyl-1,2,3,4,4a,9,10,10a-octahydrophenanthrene (3.6%), 3,6-dimethylphenanthrene (2.9%) and 2,3,5-trimethylphenanthrene (3.2%)].

**Scheme 1:**
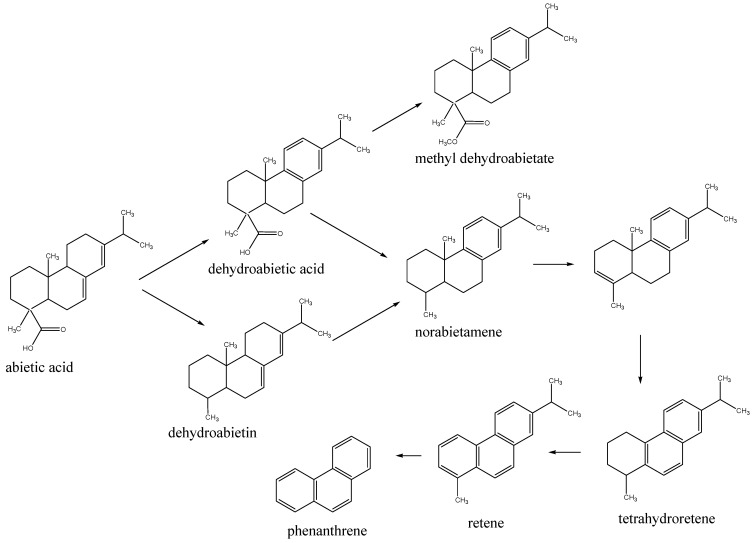
Transformation reactions of abietic acid.

Abietic acid is the main component in resins of *Pinaceae* origin [5]. During the heating process abietic acid (AA) can be converted into dehydroabietic acid (DHA) through dehydrogenation [20]. A series of other transformations can produce many other intermediate organic compounds when the thermal treatment is maintained and is more intense (Scheme 1). Although the main reaction is dehydrogenation of AA to DHA, decarboxylation of DHA produces dehydroabietin. Increasing aromatization of dehydroabietin as well as decarboxylation of DHA generates norabietamene. Norabietamene is further dehydrogenated to tetrahydroretene and retene (formed predominantly when the process is carried out at high temperatures [20]). Further retene dealkylation produces phenathrene.

Methyl DHA is formed when the resin is heated in the presence of wood because CH_3_OH released when wood is heated to high temperatures reacts easily with DHA, which is absent when the sealing material is produced by pyrolysis of the resin alone [2,20]. The simultaneous presence of retene and methyl dehydroabietate highlights that the resin was heated in the presence of wood obtained from plants of the *Pinaceae* family [2,20]. 

In general, oxygenated products (such as 7-oxo-DHA or 7-oxo-15-hydroxy-DHA) are the consequence of an aging process due to contact with the atmosphere. These compounds were abundant in other ancient organic residues that were GC analyzed after derivatization [3,21]. Direct GC analysis of the sample is limiting for determination of such high-molecular weight oxygenated products. However, the organic residue was preserved under the sea for a long period and therefore oxygenation reactions were probably not promoted.

## 3. Experimental

### 3.1. The Samples of the Ancient Organic Residue and Pine Resin

The ancient organic residue was obtained from the bottom of a Greco-Italian amphora type Benoit Republicaine-II/Lamboglia (Figure 1a) [22] found in the Adriatic Sea (near Vis Island). These amphorae were produced from the end of the middle of the 2nd century BC until the end of the 1st century AC in the ancient Roman province of Campania [1]. The organic residue was *ca*. 3 cm high and 7 cm in length. The sample for this research was taken from the inner part to avoid potential contaminants from its external surface. The sample of pine resin (10 g) was collected from *Pinus* trees from the Adriatic coast of Croatia.

### 3.2. Headspace Solid-Phase Microextraction (HS-SPME)

The isolation of headspace volatiles was performed using a manual SPME fibre with a layer of polydimethylsiloxane/divinylbenzene (PDMS/DVB) as well as a fibre with a layer of divinylbenzene/carboxene/polydimethylsiloxane (DVB/CARPDMS) obtained from Supelco Co. (Bellefonte, PA, USA). The fibre was conditioned prior to use according to the manufacturer’s instructions. For HS-SPME extraction, the samples (fine powered residue and pine resin) were placed in a 15 mL glass vial and hermetically sealed with PTFE/silicone septa. The vial was maintained in a water bath at 60 °C during equilibration (20 min) and extraction (45 min). After sampling, the SPME fibre was withdrawn into the needle, removed from the vial, and inserted into the injector (250 °C) of the GC and GC-MS for 6 min where the extracted volatiles were thermally desorbed directly onto the GC column.

### 3.3. Dissolution of the Samples in CH_2_Cl_2_

A part of the organic residue (15 mg) was dissolved in CH_2_Cl_2_ (2 mL) and 1 μL was used for direct GC and GC-MS analyses. Pine resin was dissolved like the ancient organic residue.

### 3.4. Gas Chromatography and Mass Spectrometry (GC, GC-MS)

Gas chromatography analyses were performed on an Agilent Technologies (Palo Alto, CA, USA) gas chromatograph model 7890A equipped with a flame ionization detector, mass selective detector, model 5975C and capillary column HP-5MS [(5%-phenyl)-methylpolysiloxane Agilent J & W GC column, 30 m, 0.25 mm i.d., coating thickness 0.25 μm]. Chromatographic conditions were as follows: helium was carrier gas at 1 mL·min^−1^, injector temperature was 250 °C, and FID detector temperature was 300 °C. HP-5MS column temperature was programmed at 70 °C isothermal for 2 min, and then increased to 200 °C at a rate of 3 °C·min^−1^ and held isothermal for 18 min. The injected volume was 1 μL and the split ratio was 1:50. MS conditions were: ionization voltage 70 e.V.; ion source temperature 230 °C; mass scan range: 30–300 mass units. The analyses were carried out in duplicate.

### 3.5. Data Analysis and Data Evaluation

The individual peaks were identified by comparison of their retention indices (relative to C_9_-C_25_ *n*-alkanes for HP-5MS) to those of authentic samples (lower aliphatic alcohols, carbonyls, acids and esters; benzene derivatives; monoterpenes; retene; methyl dehydroabietate; abietic acid; 55 compounds from the organic residue were identified by comparison with the standards and 24 compounds from the resin) and literature [23], as well as by comparing their mass spectra with the Wiley 275 MS library (Wiley, NY, USA) and NIST02 (Gaithersburg, MD, USA) mass spectral database. The percentage composition of the samples was computed from the GC peak areas using the normalization method (without correction factors). The component percentages (Table 1 and Table 2) were calculated as mean values from duplicate GC and GC-MS analyses.

## 4. Conclusions

Direct GC and GC-MS analysis (without derivatization) of the ancient organic residue from a Roman amphora revealed possible chemical traces of trapped wine aroma (low-molecular weight aliphatic alcohols, acids, esters and carbonyls with 2-phenylethanol) in the headspace chemical profile. Derivatization procedures from previous papers, although they have provided insight into the major high-molecular weight compounds of amphora residues, they have not revealed the low-molecular weight compounds that can be important chemical tracers of the amphora’s original content. The dhemical profile of the dissolved residue was dominated by methyl dehydroabietate and retene, important chemical markers of the pine source and preparation procedure of the water-proof coating along with phenanthrene and naphthalene derivatives.

## Figures and Tables

**Table 1:** The organic residue VOC composition obtained by HS-SPME and from the residue CH_2_Cl_2_ solution after direct GC and GC-MS analysis.

No.	Compound	RI	A	B	C
1.	Acetone	< 900	0.3	2.8	-
2.	Acetic acid	< 900	0.1	2.0	-
3.	Butan-2-one	< 900	-	0.6	-
4.	Ethyl acetate	< 900	-	0.4	-
5.	Benzene	< 900	-	0.2	-
6.	Propionic acid	< 900	0.1	2.1	-
7.	Heptane	< 900	-	0.7	-
8.	Ethyl propionate	< 900	-	0.4	-
9.	Isoamyl alcohol	< 900	0.3	0.9	-
10.	Toluene	< 900	-	0.5	-
11.	Butanoic acid	< 900	-	1.6	-
12.	Octane	< 900	-	1.6	-
13.	Furfural	< 900	-	0.5	-
14.	Isovaleric acid	< 900	-	0.4	-
15.	Ethylbenzene	< 900	-	0.4	-
16.	Isoamyl acetate	< 900	0.1	0.8	-
17.	*p*-Xylene	< 900	-	0.8	-
18.	Valeric acid	< 900	-	1.2	-
19.	Heptan-2-one	< 900	-	1.0	-
20.	Nonane	900	-	0.6	-
21.	Heptanal	905	-	0.4	-
22.	Isopropylbenzene (cumene)	932	-	0.4	-
23.	α-Pinene	941	0.1	0.2	-
24.	Camphene	958	0.2	0.4	-
25.	Benzaldehyde	965	0.6	20.4	-
26.	Hexanoic acid (caproic acid)	994	0.1	4.6	-
27.	Octanal	1007	-	0.7	-
28.	*p*-Cymene	1032	0.1	2.7	-
29.	Limonene	1036	-	0.5	-
30.	2-Hydroxybenzaldehyde (salicylaldehyde)	1052	-	0.2	-
31.	1-Phenylethanone (acetophenone)	1074	-	0.7	-
32.	Heptanoic acid	1093	-	0.8	-
33.	Fenchone	1094		0.2	
34.	Nonan-2-one	1097	-	1.3	-
35.	1-Ethyl-4-(1-methylethyl)-benzene	1110	-	1.6	-
36.	2-Phenylethanol	1116	1.2	3.3	-
37.	Camphor	1153	-	0.9	-
38.	Pentylbenzene	1165	-	0.4	-
39.	Borneol	1175	1.1	4.5	0.2
40.	Naphthalene	1190	-	1.2	-
41.	Octanoic acid	1191	0.5	1.4	-
42.	2-Methoxy-4-methylphenol (*p*-cresol)	1198	0.3	0.9	-
43.	3-Isopropylbenzaldehyde	1229	0.4	4.7	-
44.	4-Ethyl-2-methoxyphenol (4-ethylguaiacol)	1286	0.4	0.9	-
45.	Nonanoic acid	1288	-	0.5	-
46.	Bornyl acetate	1291	-	0.6	-
47.	1-Methylnaphthalene	1316	-	0.4	-
48.	*p*-Acetylacetophenone	1325	-	0.7	-
49.	2-Methoxy-4-propylphenol	1375	1.2	1.5	-
50.	4-Hydroxy-3-methoxy-benzaldehyde (vanillin)	1406	0.3	-	-
51.	1,7-Dimethylnaphthalene ^**^	1427	0.4	0.7	-
52.	3,5-Dimethoxybenzaldehyde (syringaldehyde)	1445	0.3	0.5	-
53.	4-Hydroxy-3-methoxy-acetophenone (acetovanillone)	1494	0.3	-	-
54.	α-Muurolene	1507	0.6	0.9	-
55.	Calamenene	1531	1.0	1.3	-
56.	γ-Cadinene	1535	0.1	-	-
57.	α-Calacorene	1551	0.1	-	-
58.	2,3-Dimethylbenzofuran	1578	0.3	-	-
59.	1-Methyl-7-(1-methylethyl)-naphthalene (eudalin)	1587	0.6	0.5	-
60.	1,6,7-Trimethylnaphthalene ^*^	1590	0.3	-	-
61.	1,6-Dimethyl-4-(1-methylethyl)-naphthalene (cadalin)	1684	1.1	0.7	-
62.	3,4-Diethyl-1,1′-biphenyl	1751	0.4	-	-
63.	Anthracene	1780	0.1	-	-
64.	4-(1-Methyl-1-phenyl)-phenol (4-cumylphenol)	1814	0.8	-	-
65.	Cembrene ^**^	1912	2.0	0.7	-
66.	2-Methylanthracene ^**^	1925	1.8	0.5	0.3
67.	1-Methoxy-8-methylnaphthalene ^*^	1942	2.2	-	0.3
68.	Hexadecanoic acid	1963	-	-	0.9
69.	7-(1-Methylethyl)-1,4a-dimethyl-1,2,3,4,4a,9,10,10a-octahydro-naphthalene	1988	9.3	2.7	2.8
70.	7-(1-Methylethyl)-1,4a-dimethyl-2,3,4,4a,9,10-hexahydro-phenanthrene	1996	7.7	1.0	1.9
71.	7-(1-Methylethyl)-1,4a-dimethyl-1,2,3,4,4a,9,10,10a-octahydro-phenanthrene	2023	12.2	3.4	3.6
72.	3,6-Dimethylphenanthrene ^**^	2049	6.6	1.0	2.9
73.	1,4-Dimethoxyphenathrene ^*^	2103	10.0	1.7	6.8
74.	2,3,5-Trimethylphenanthrene	2167	1.2	-	3.2
75.	7-Ethenyl-1,2,3,4,4a,4b,5,6,7, 9,10,10a-dodecahydro-1,4a,7-trimethyl-1-phenanthrene-carboxaldehyde (pimaral)	2187	0.6	-	0.8
76.	Methyl 8,15-pimaradien-18-oate	2227	0.6	-	0.8
77.	1-Methyl-7-(1-methylethyl)-phenanthrene (retene)	2242	9.9	1.6	24.1
78.	Methyl 8,15-isopimaradien-18-oate	2256	0.5	-	0.6
79.	4-(2-Ethyl-5-phenyl-1H-pyrrol-3-yl)-pyridine ^*^	2382	0.7	-	4.2
80.	Methyl 6,8,11,13-abietatetraen-18-oate ^*^	2451	-	-	1.7
81.	Methyl dehydroabietate	2477	6.0	1.0	33.6
	Total identified (%)		85.1	94.7	88.7

RI = retention indices on HP-5MS column; **A** = solvent-free HS-SPME with the fiber PDMS/DVB; **B** = solvent-free HS-SPME with the fiber DVB/CARPDMS; **C** – the residue dissolved in CH_2_Cl_2_; - = not identified; ^*^ – tentatively identified; ^**^ – correct isomer not identified.

**Table 2:** The pine resin VOCs composition obtained by HS-SPME and from the resin CH_2_Cl_2_ solution after direct GC and GC-MS analysis.

No.	Compound	RI	A	B	C
1.	Toluene	< 900	0.3	3.2	-
2.	α-Pinene	941	73.4	66.2	52.0
3.	Camphene	958	2.1	3.8	4.2
4.	Verbenene	961	0.6	0.7	3.5
5.	Benzaldehyde	965	-	0.2	-
6.	β-Pinene	983	1.0	0.9	0.3
7.	Myrcene	994	0.4	0.2	-
8.	Δ-3-Carene	1016	0.3	0.2	-
9.	*p*-Cymene	1032	0.5	1.4	0.9
10.	Limonene	1035	2.9	4.4	1.5
11.	α-Terpinolene	1093	0.3	0.3	-
12.	*p*-Cymenyl	1095	0.4	2.3	1.5
13.	Fenchol	1123	0.1	0.2	0.7
14.	α-Campholene aldehyde	1133	0.4	0.4	0.9
15.	*trans*-Pinocarveol	1148	0.3	0.4	1.0
16.	Camphor	1153	0.2	0.1	0.3
17.	Borneol	1175	0.2	0.3	3.8
18.	Terpinen-4-ol	1185	0.1	0.1	-
19.	*p*-Cymen-8-ol	1194	0.2	0.4	0.6
20.	α-Terpineol	1198	0.7	1.1	2.3
21.	Estragole	1204	0.8	0.9	-
22.	Verbenone	1217	0.4	0.6	2.4
23.	β-Caryophyllene	1425	5.7	2.0	3.0
24.	β-Selinene	1460	1.1	0.4	-
25.	α-Muurolene	1506	2.3	1.6	2.0
26.	Caryophyllene oxide	1589	0.8	0.2	-
27.	Methyl dehydroabietate	2477	-	-	2.7
	Total identified (%)		95.5	92.5	83.6

RI = retention indices on HP-5MS column; **A** = solvent-free HS-SPME with the fiber PDMS/DVB; **B** = solvent-free HS-SPME with the fiber DVB/CARPDMS; **C** – the residue dissolved in CH_2_Cl_2_; - = not identified; ^*^ – tentatively identified; ^**^ – correct isomer not identified.
